# Safety of Intracoronary Infusion of 20 Million C-Kit Positive Human Cardiac Stem Cells in Pigs

**DOI:** 10.1371/journal.pone.0124227

**Published:** 2015-04-23

**Authors:** Matthew C. L. Keith, Xian-Liang Tang, Yukichi Tokita, Qian-hong Li, Shahab Ghafghazi, Joseph Moore IV, Kyung U. Hong, Brandon Elmore, Alok Amraotkar, Brian L. Ganzel, Kendra J. Grubb, Michael P. Flaherty, Gregory Hunt, Bathri Vajravelu, Marcin Wysoczynski, Roberto Bolli

**Affiliations:** 1 Institute of Molecular Cardiology, University of Louisville, Louisville, Kentucky, United States of America; 2 Department of Cardiothoracic Surgery, University of Louisville, Louisville, Kentucky, United States of America; Georgia Regents University, UNITED STATES

## Abstract

**Background:**

There is mounting interest in using c-kit positive human cardiac stem cells (c-kit^pos^ hCSCs) to repair infarcted myocardium in patients with ischemic cardiomyopathy. A recent phase I clinical trial (SCIPIO) has shown that intracoronary infusion of 1 million hCSCs is safe. Higher doses of CSCs may provide superior reparative ability; however, it is unknown if doses >1 million cells are safe. To address this issue, we examined the effects of 20 million hCSCs in pigs.

**Methods:**

Right atrial appendage samples were obtained from patients undergoing cardiac surgery. The tissue was processed by an established protocol with eventual immunomagnetic sorting to obtain *in vitro* expanded hCSCs. A cumulative dose of 20 million cells was given intracoronarily to pigs without stop flow. Safety was assessed by measurement of serial biomarkers (cardiac: troponin I and CK-MB, renal: creatinine and BUN, and hepatic: AST, ALT, and alkaline phosphatase) and echocardiography pre- and post-infusion. hCSC retention 30 days after infusion was quantified by PCR for human genomic DNA. All personnel were blinded as to group assignment.

**Results:**

Compared with vehicle-treated controls (n=5), pigs that received 20 million hCSCs (n=9) showed no significant change in cardiac function or end organ damage (assessed by organ specific biomarkers) that could be attributed to hCSCs (P>0.05 in all cases). No hCSCs could be detected in left ventricular samples 30 days after infusion.

**Conclusions:**

Intracoronary infusion of 20 million c-kit positive hCSCs in pigs (equivalent to ~40 million hCSCs in humans) does not cause acute cardiac injury, impairment of cardiac function, or liver and renal injury. These results have immediate translational value and lay the groundwork for using doses of CSCs >1 million in future clinical trials. Further studies are needed to ascertain whether administration of >1 million hCSCs is associated with greater efficacy in patients with ischemic cardiomyopathy.

## Introduction

C-kit^pos^ cardiac stem cells (CSCs) are one of a number of stem/progenitor cells described in the mammalian heart and one of the two types ever used clinically for cardiac regeneration [1,2]. We recently reported the results of the first in-human clinical trial of autologous c-kit^pos^ CSCs in patients with ischemic cardiomyopathy [2]. In this phase I trial, designed to evaluate the safety and feasibility of intracoronary administration of c-kit^pos^ CSCs, 1 million cells were injected in the infarct-related artery using the stop-flow technique. The administration of c-kit^pos^ CSCs was shown to be safe and there were encouraging results related to efficacy, with a significant improvement in left ventricular (LV) ejection fraction in the hCSC treated group [2]. These encouraging findings have sparked growing interest in utilizing c-kit^pos^ CSCs in additional trials with escalating doses >1 million cells, particularly in light of reports of dose-dependent responses with stem cells[3]. However, the safety of higher doses of c-kit^pos^ CSCs has never been evaluated in any clinical or preclinical model.

Intracoronary administration has been utilized with many cell types. Mesenchymal stromal cells (MSCs) have been used in a large number of cardiac regeneration trials. Although these cells are usually administered via direct transendocardial injection, a number of clinical trials have used them intracoronarily [4–7]. The cell dose in these studies ranged from 1 million to > 100 million cells [4,5,7–9]. A number of other studies are ongoing with intracoronary administration of MSCs (e.g., RELIEF-NCT01652209). Nevertheless, there still exist important safety concerns with intracoronary injection of MSCs [10–12]. Grieve et al. demonstrated that although intracoronary infusion of 25 million MSCs was safe, 75 million cells caused biochemical and histological myocardial infarction in an ovine model [11]. Similarly, Vulliet et al. showed a dose-dependent rise in ST segments during intracoronary injection of MSCs in all 7 dogs studied [12]. The average size of MSCs and BMMNCs is ~21.0±3.3 μm and 8.6±1.8 μm, respectively [13]. At 7 to 10 μm, the typical capillary luminal diameter is smaller than the average sized MSCs, the likely explanation for the findings in the aforementioned studies [13,14]. In the CADUCEUS trial, 12.5 to 25 million cardiosphere-derived cells (CDCs) were injected intracoronarily with no significant safety concern [15]. However, administration of 50 million allogeneic CDCs resulted in large infarctions in pigs [13]. Measuring 20.6±3.9 μm in diameter, CDCs are larger than the average capillary diameter, thereby causing microvascular obstruction in that model. Finally, bone marrow mononuclear cells (BMMNCs) have almost always been administered intracoronarily. Close to 100 phase I and II clinical trials using large numbers of BMMNCs have demonstrated that intracoronary administration is safe [16]. For instance, within the three phase II trials of BMMNCs led by the Cardiovascular Cell Therapy Research Network (CCTRN), 100–150 million cells were administered intracoronarily with no complications [17,18].

C-kit^pos^ CSCs are similar in size to the unselected cell population from which they are sorted, ranging from ~12 to 20 μm in diameter in suspension. Therefore, it is conceivable that they could bring about significant microvascular obstruction if administered in high enough doses. Therefore, we set out to investigate the safety of 20 million intracoronarily delivered c-kit^pos^ human CSCs, a dose ~40 times higher than that used in our previous porcine study[19], in a porcine model, as a preamble to future clinical trials.

## Methods

**Fig 1:**
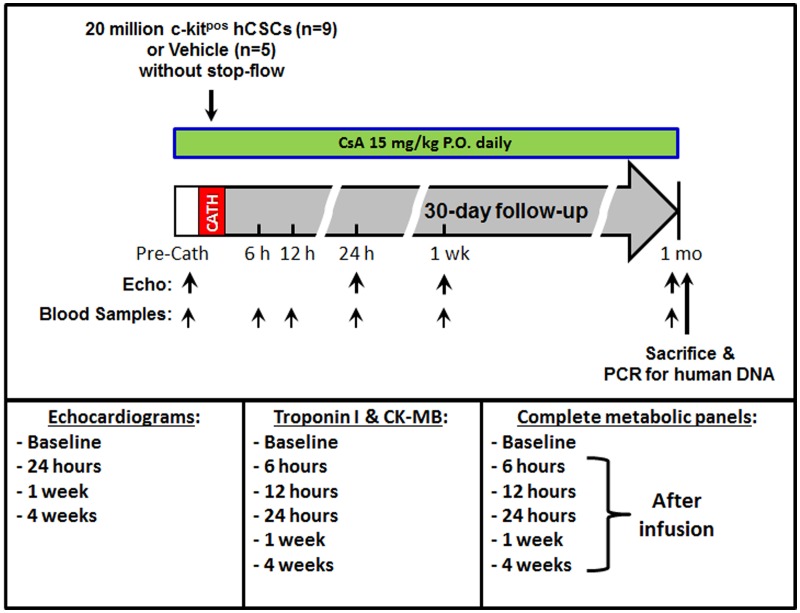
Study protocol and timeline.

A detailed timeline of the experimental protocol is illustrated (Fig 1). Detailed datasets can be found in the supplemental supporting information, specifically S1–S10 Tables.

### Human c-kit^pos^ CSC isolation and expansion

**Fig 2:**
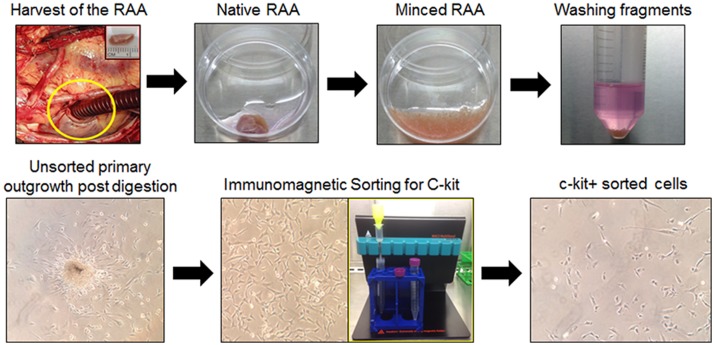
Isolation and expansion of c-kit^pos^ hCSCs. Right atrial appendages (RAA) were harvested with subsequent mechanical and enzymatic digestion to obtain primary outgrowth of total adherent cardiac cells. Primary cells were immunomagnetically sorted for c-kit and the resultant cells expanded *in vitro*.

Right atrial appendage specimens were obtained with IRB approval (IRB number 07.0062) from patients undergoing open-heart, on pump, coronary artery bypass surgery at Jewish Hospital in Louisville, Kentucky. All patients were between the ages of 50 and 75 years of age, so as to approximate the ages of patients that were included in the recently conducted SCIPIO phase I clinical trial[2]. Right atrial appendages were transported to the cell processing lab under sterile conditions on wet ice. The tissue was washed several time with ice cold PBS to remove gross blood. Adipose tissue was then resected manually from the external surface of the tissue with subsequent repeated washing in cold PBS. The tissue was then manually minced to obtain fragments < 1mm3 (Fig 2). The tissue fragments were then incubated on a shaking incubator at 37°C in Worthington Collagenase type II/Hams F12 solution with multiple rounds enzymatic digestion. Once complete, the solution of released cells was centrifuged with discarding of the supernatant. The cells were washed in full growth media consisting of Ham’s F12 (Gibco), 10% FBS (Thermo Scientific Hyclone), 10ng/ml Recombinant Human bFGF (PeproTech), 0.2mM L-Glutathion (Sigma), human Erythropoietin (Sigma), and 100U/ml penicillin/streptomycin (Gibco). The supernatant was discarded and the cells were resuspended in full growth media and plated in a 6-well plate for passage 0 initial expansion. Media was changed at 24 h completely. Additional media changes were performed every 3–4 days or if necessitated by visual examination of the culture. Cells were expanded until 70% confluence at which time they were passaged to T75 Flasks for additional subconfluent expansion prior to immunoselection for c-kit expression. Media was added or changed partially every 3–4 days for the remainder of the culture process. Cells were passaged 1 time prior to immunomagnetic sorting for c-kit (CD117) using Miltenyi immunomagnetic beads according to manufacturer’s recommendations. Illustrations of the tissue processing and initial cell expansion are shown in Fig 2.

### c-kit^pos^ hCSC immunomagnetic sorting (MACS)

Passage 1 cells at 70–75% confluence in T75 culture flasks were sorted for c-kit with anti-CD117 Miltenyi microbeads and Miltenyi magnetic sorting apparatus (Fig 2). Cell sorting was performed through the direct technique. Cells were trypsined and washed twice in ice cold MACS buffer made per manufacturer’s specifications. All solutions were cooled on ice prior to beginning the sorting protocol. Cells were immunomagnetically sorted according to manufacturer’s specifications using Miltenyi MS columns and pre-separation filters with magnetic stand. Positively selected cells were plated in 6-well plates at subconfluence for subsequent *in vitro* expansion of c-kit^pos^ cells (Fig 2). Human c-kit^pos^ CSCs were expanded exponentially over 3–4 additional passages to ultimately obtain approximately 3 x 10^7^ cells per patient. Multiple patients cells were pooled to obtain a uniform cell product that was ultimately infused intracoronarily into the treatment group of pigs (n = 9). Cells were assessed by flow cytometric analysis per standard protocol for c-kit positivity at passage 3–4. Only populations of cells showing greater than 70% c-kit positivity were used for the study.

### Flow cytometric analysis and immunocytochemistry

**Fig 3:**
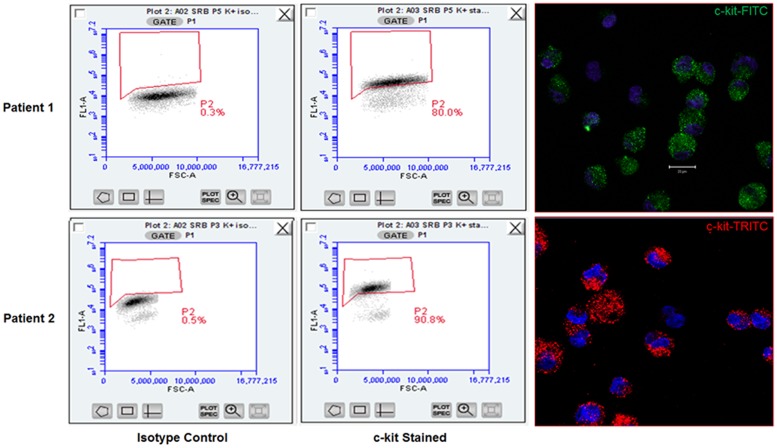
Flow cytometric validation and immunocytochemistry of c-kit^pos^ hCSCs. Representative flow cytometric analyses of isotype control (left) and c-kit-labeled cell flow plots (center) are shown. Suspension immunocytochemistry of c-kit^**pos**^ hCSCs showing positive anti-c-kit labeling is shown in the right panels, with DAPI labeled nuclei in blue.

**Fig 4:**
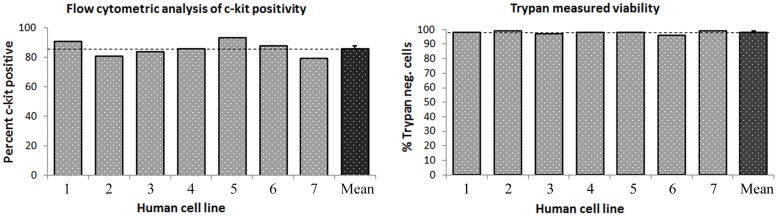
Cumulative c-kit positivity by flow cytometry and Trypan blue cell product viability. The left panel shows c-kit positivity in seven cell lines utilized for the study, which averaged 85.6%±1.9% (mean±SEM). The right panel shows viability of c-kit^**pos**^ hCSCs measured by cellular exclusion of Trypan blue staining prior to intracoronary infusion. Trypan negative, viable cells averaged 97.8±0.4%. Data are mean±SEM.

Cells were trypsinized from dedicated flasks at passage 3–4 per standard protocol. Cells were washed 1x in ice cold buffer with 1% bovine serum albumin (BSA)/phosphate buffered saline (PBS) buffer followed by a second wash in cold PBS. Cells were then fixed with 15 minutes at room temperature in freshly prepared or commercially purchased 4% PFA buffered to pH 7. Fixed cells were washed twice in PBS. Cells were stained for c-kit directly after fixation. Cells were blocked for 10 minutes at RT in 1% BSA buffer and then stained for c-kit with c-terminal specific Santa Cruz C19 rabbit polyclonal IgG anti-human c-kit antibody for 1 h at room temperature in the dark. Isotype rabbit polyclonal IgG in identical concentration was used in parallel as an isotype control. Cells were then washed twice with 1% BSA buffer. Secondary antibody, FITC or TRITC conjugated Invitrogen Donkey anti-rabbit IgG was then added for 1 h at room temperature in the dark for flow cytometry or confocal microscopic imaging after cells were spun onto glass slides. Confocal images were taken using Zeiss 510 inverted confocal microscope and image processing performed relative to isotype control labeling with integral instrument software only. Flow cytometric analysis was performed using BD Accuri C6 flow cytometer. All analysis gates were set for false positivity of <1% in respective isotype controls (Fig 3). Accuri C6 software was used for final analysis of c-kit positivity. Illustrations of flow plots and immunocytochemistry images (Fig 3) as well as data regarding c-kit positivity of all cell lines utilized for the study are shown (Fig 4). Only cell lines with greater than 70% c-kit positivity measured by flow cytometric analysis were utilized for the study.

### Cell product generation

Positively selected cells were expanded *in vitro* for 3–4 additional passages prior to intracoronary infusion. *In vitro* expanded c-kit^pos^ CSCs were trypsinized and washed with sterile PBS and resuspended in 12mL of Plasmalyte-A solution. Final cell count and viability by hemocytometer and Trypan blue were performed. Cell number was adjusted by volume to closely approximate 20 million cells in 12mL Plasmalyte-A solution. The cells were placed on wet ice and transported to the cath lab for intracoronary infusion.

### Ethics statement

This study was carried out in strict accordance with the recommendations in the Guide for the Care and Use of Laboratory Animals of the National Institutes of Health. The protocol was approved by the Institutional Animal Care and Use Committee (IACUC) of the University of Louisville (IACUC number: 12114).

### Animal procedures

Female Yorkshire pigs (weight 32.4 ± 0.7 kg, age: 13.4 ± 0.3 weeks) were used for this study. All animal procedures were approved by the University of Louisville IACUC (IACUC number 12114) prior to initiation of the study. Pigs were fasted for at least 12 h prior to sedation. On the day of cell delivery, pigs received a prophylactic dose of antibiotics (Ceftiofur 3 mg/kg, IM) and a preemptive dose of analgesics (buprenorphine 0.025 mg/kg, IM). Pigs were sedated using a cocktail of ketamine (20 mg/kg, i.m.) and xylazine (2 mg/kg, i.m.). An intravenous catheter was placed in a marginal ear vein for the administration of fluids and drugs. Animal received diazepam (1 mg/kg IV) to facilitate intubation. Following adequate sedation, pigs were intubated and mechanically ventilated. General anesthesia was maintained with isoflurane (1.5%- 2.0% 50/50 oxygen/nitrogen). Pigs received aspirin (2300 mg, IV) and heparin (300 U/kg, IV) before the catheterization procedure.

A cut-down on the right neck was performed and the right jugular was used for placement of a 7–10 F chronic cath polyurethane catheter (Access Technologies or Bard/Hickman). This catheter was implanted, secured, tunneled to the back of the neck and kept in place for the duration of the 1 month follow-up for serial blood collections; using this catheter, blood samples were obtained for serial measurement of cardiac markers at baseline (before catheterization procedure) and at 6, 12, 24 h, and 1 week, and 1 month after cell delivery (Fig 1). The catheter dead-space was measured and filled with heparin (1000 units/mL) after each withdrawal to maintain patency. Care was taken to fill the dead-space only with no spillover into the systemic circulation after each blood collection.

At the end of the 1 month follow up, pigs were anesthetized with 22 mg/kg ketamine and 2 mg/kg xylazine IM. Pig was transported to the cath lab for final hemodynamic measurement. Animals received diazepam 1 mg/kg IV to facilitate intubation. Animals were then again intubated and ventilated. General anesthesia was maintained with isoflurane (1.5%- 2.0% 50/50 oxygen/nitrogen). Hemodynamic variables were monitored and recorded. After the final hemodynamic recording was taken, the animal was deeply anesthetized with 5% isoflurane. A bolus of 3–6 ml/kg of 3 mmol/ml potassium chloride solution was injected intravenously until the heart was completely arrested. Asystole was confirmed by cessation of cardiac electric activity from ECG monitoring. The chest was opened via a left thoracotomy, the aorta was transected, and the pig exsanguinated. The heart was then harvested.

### Cell delivery / catheterization procedure

Through a right femoral artery cut-down, a 7F fast-cath sheath was introduced. A 6 F Hockey-stick catheter (Cordis) was fluoroscopically guided to the left main coronary artery. The left main coronary ostium was engaged by the catheter and an angioplasty-type balloon catheter (Maverick 2.0 x 9 mm) and guide wire (BMW, Boston Scientific) assembly was guided into the LAD; the wire was advanced into the mid LAD and the catheter telescoped over the wire and positioned just proximal to the 1st diagonal branch. The CSC solution (20 million cells in 12 ml of sterile Plasma-Lyte A solution or vehicle, divided by 4 injections, 3 ml each, interspersed with 4’30” between each injection) or vehicle (12 ml of sterile Plasma-Lyte A solution) was injected manually at a constant rate through the central port of the angioplasty balloon catheter over the 3 min. After the procedure, the Hockey-stick catheter, and the femoral sheath were removed and the groin access site was closed in 3 layers using 3–0 PDS suture. A transdermal fentanyl patch (2.5 μg/kg/h) was placed at the end of the procedure for postoperative analgesics. The pigs were weaned from anesthesia, extubated, and moved to a post-operative area for postoperative monitoring. Ceftiofur (3 mg/kg, s.c.) was be repeated on day 1 and 2 post-procedure.

Immunosuppressive Therapy: Pigs received 15 mg/kg/day of Cyclosporine A (CsA) starting 2 days before cell injection and continuing until the end of follow-up. CsA (powder from Novartis) was mixed with a tablespoon of grape flavored Kool-Aid powder and ~60 ml of drinking water to make a suspension beverage to feed the animal orally.

### Echocardiography

Echocardiograms were obtained at baseline (before CSC delivery), 24 h, 1 week, and 1 month after CSC delivery (Fig 1) using a HP SONOS 7500 ultrasound system (Philips Medical Systems) equipped with a HP 21350A (S8) 3.0–8.0 MHz sector array ultrasound transducer. Before the echocardiographic study, pigs were anesthetized (isoflurane) and placed in the left lateral decubitus position. Temperature was monitored with a rectal temperature probe and kept between 37.0°C and 37.5°C with a heating pad. The parasternal short-axis view was used to obtain 2D and M-mode images19. Systolic and diastolic anatomic parameters were obtained from M-mode tracings at the mid-papillary level. Digital images were analyzed off-line by a single blinded observer using ComPACS Review Station (version 10.5) image analysis software (Medimatic, Las Cruces, NM 88004, USA) according to the American Society of Echocardiography standards[20].

### Cardiac biomarkers assays: Troponin I and CK-MB

Plasma cTnI levels were measured with a pig cTnI ELISA kit according to the manufacturer’s instruction (Life Diagnostics, West Chester, PA) at baseline as well as 6, 12, and 24 hrs, and one week and one month post infusion (Fig 1). Fresh blood was collected with the heparinized tube and then centrifuged to separate plasma from the blood sample. Each assay was performed in duplicate and in a blinded fashion. The original cTnI data (i.e., the optical density absorbance values acquired from Beckman Coulter DU730 Spectrophotometer) were calibrated and converted with cTnI standard curve. The final cTnI results were expressed as nanogram per milliliter plasma (ng/ml). All measurements were performed in duplicate.

Plasma CK-MB concentration was measured via an ELISA kit (MyBioSource) at baseline as well as 6, 12, and 24 hrs, and one week and one month post infusion (Fig 1). Briefly, the 96-well plate was pre-coated with an antibody specific to pig CK-MB. The plasma samples were loaded onto the wells and bound by the pre-coated specific antibody. Then a biotinylated detection antibody specific for pig CK-MB and Avidin-Horseradish Peroxidase (HRP) conjugate were added to the wells. After incubation, free components were washed away. The substrate solution was added to each well. Only the wells contained CK-MB protein, biotinylated detection antibody and Avidin-HRP conjugate presented blue in color. This enzyme-substrate reaction was terminated by the addition of a sulphuric acid solution, therefore the color turned to yellow. The optical density (OD) was measured spectrophotometrically at a wavelength of 450 nm. The original CK-MB data (i.e., OD values) in the pig plasma samples were calculated and converted with pig CK-MB standard curve. Duplicate assays were performed for each sample and CK-MB concentration was expressed as nanogram per milliliter of plasma (ng/ml). All CK-MB assays and calculation were conducted in a blinded fashion relative to hCSC treated vs control group. All measurements were performed in duplicate.

### Assessment of renal and hepatic function

Functions were assessed by measurement of respective biomarkers. Peripheral blood was drawn and complete metabolic panels (CMPs) were obtained in hCSC-treated and control groups at baseline and 6, 12, and 24 h, 1 week, and 1 month after intracoronary infusion (Fig 1). All CMPs were analyzed by the veterinary lab company *Antech Diagnostics* (Louisville, Ky) in a blinded fashion. Resultant biomarker values were compared with known porcine reference ranges provided by *Antech Diagnostic* as well as with absolute initial baseline values. Changes in blood urea nitrogen (BUN) and creatinine (Cr) were used to assess renal function. Change in AST, ALT, alkaline phosphatase (Alk Phos), and total creatine phosphokinase (CPK) were used to assess hepatic function.

### DNA isolation from paraffin embedded sections and PCR

Genomic DNA was isolated from representative paraffin embedded left ventricular tissue sections using QIAamp DNA FFPE Tissue Kit (Qiagen) according to the manufacturer’s instructions. Samples were analyzed for the presence of human (HLA-DMA) and pig (Pig Gapdh) genomic DNA using the following primer sets:

- HLA-DMA fwd, 5’-TACAAACCTCAGCTACCTTCGTGGC-3’
- HLA-DMA rev, 5’-AACCCAGCTGACTCTGGGTGG-3’
- Pig Gapdh fwd, 5’-CCCCCTCAGATTTGGCCGCA-3’
- Pig Gapdh rev, 5’-CACGGGGGCCACTCACCAT-3’

For PCR reaction, 100 ng of each DNA sample was amplified in a 20 μl reaction for 40 cycles (denaturation at 95°C; annealing at 61°C; and extension at 72°C) using Taq 2X Master Mix (New England Biolabs). Accordingly, the limiting threshold or sensitivity of detection of human CSCs approximated 1 hCSC per 15,000 porcine cells.

### Statistical analyses

One or two-way repeated measures ANOVA statistical analysis was employed for all comparisons of echocardiographic parameters as well as cardiac, hepatic, and renal biomarkers between groups across multiple time points where applicable. All data are represented as means ± SEM. Datasets are illustrated in the supplemental supporting information.

## Results

### Intracoronary infusion of 20 million c-kit^pos^ hCSCs does not impair LV function or structure

**Fig 5:**
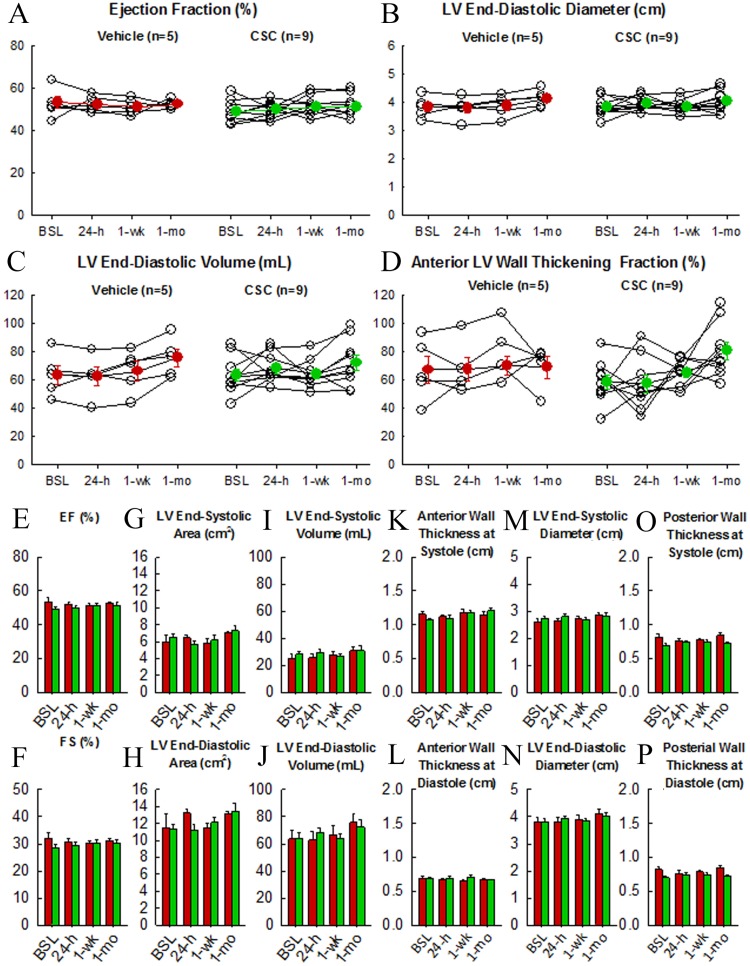
Intracoronary infusion of 20 million human c-kit^pos^ CSCs does not impair left ventricular (LV) function or morphology. The line graphs in the top panel show individual values of each pig’s progress over time (baseline, 6, 12, 24 h, 1 week, and 1 month). Individual plots are in yellow, and group means are illustrated by the red line plots. **A.** ejection fraction (EF). **B.** LV end-diastolic diameter (EDD). **C.** LV end-diastolic volume (EDV), **D.** LV anterior wall fractional thickening. The bottom panel shows group mean±SEM at each time point for respective LV functional and morphologic indices. Green and red bars indicate hCSC-treated and vehicle groups respectively. **E.** ejection fraction, **F.** fractional shortening, **G.** LV end-systolic area, **H.** LV end-diastolic area, **I.** LV end-systolic volume, **J.** LV end-diastolic volume, **K.** LV anterior wall thickness in systole, **L.** LV anterior wall thickness in diastole, **M.** LV end-systolic diameter, **N.** LV end-diastolic diameter, **O.** LV posterior wall thickness in systole, **P.** LV posterior wall thickness in diastole. Data are mean±SEM. There were no significant differences between groups with respect to any parameter at respective time points (P > 0.05).

Echocardiographic measurements were performed at serial time points after intracoronary infusion of 20 million human hCSCs (n = 9) or vehicle (n = 5) (Fig 5). Baseline parameters were not significantly different between the two groups. No significant change in left ventricular (LV) function or dimensions occurred as a result of the infusion. For example, LV ejection fraction (EF) did not differ between hCSC-treated and vehicle controls at any time point (baseline: 55.8±1.8% vs 60.3±2.9%, respectively; 24 h: 56.9±1.6% vs 59.2±1.6%; 1 week: 58.2±1.7% vs 58.0±1.6%; 1 month: 58.0±2.0 vs 59.0±1.1); changes within groups compared with baseline were also not significantly different (P>0.05). Mean anterior wall thickening fraction was examined in the treatment group and was not observed to be significantly different before (58.4±4.9%) vs 24 h after CSC infusion (57.5±6.2%), P>0.05 (Fig 5D). Similarly, there was no significant difference in LV end-diastolic diameter, fractional shortening, end-diastolic volume, end-systolic volume, end-systolic diameter and anterior and posterior wall thickness in both systole and diastole. These data indicate that the intracoronary infusion of hCSCs had no deleterious effect on LV function and dimensions.

### Intracoronary infusion of 20 million c-kit^pos^ hCSCs does not cause ischemic myocardial injury

**Fig 6:**
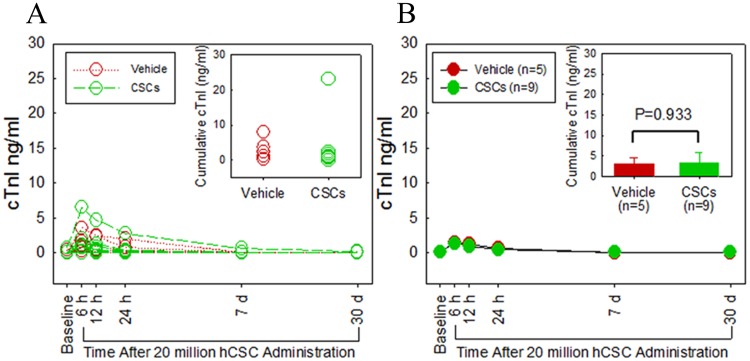
Intracoronary infusion of 20 million human c-kit^pos^ CSCs does not cause myocardial damage as assessed by cardiac troponin I (cTnI) release. **A.** Individual plots of serum cTnI levels (ng/ml) are shown at serial time points (baseline, 6, 12, 24 h, 1 week, and 1 month). Green and red plots indicate hCSC-treated and vehicle control pigs, respectively. **B.** Group means at each time point are shown. The green and red plots indicate hCSC-treated and vehicle control groups, respectively. The inset in panel B shows cumulative cTnI levels. Data are mean±SEM. There were no significant differences in plasma cTnI levels over the 1 month follow up between groups (P > 0.05 at each time point).

**Fig 7:**
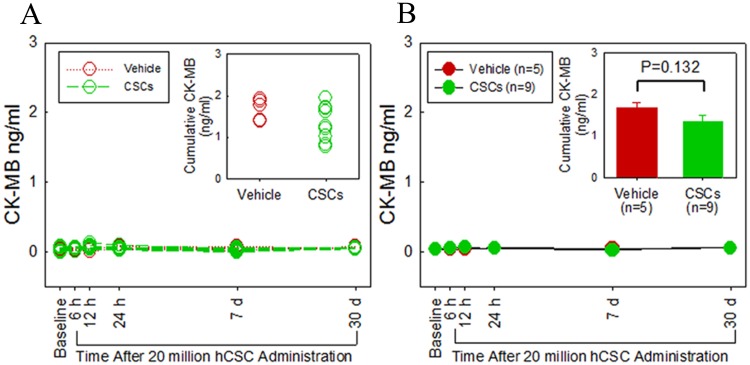
Intracoronary infusion of 20 million human c-kit^pos^ CSCs does not cause myocardial damage as assessed by cardiac CK-MB release. **A.** Individual serum CK-MB levels (ng/ml) over serial time points (baseline, 6, 12, 24 h, 1 week, and 1 month). Green and red plots identify hCSC-treated and vehicle control pigs, respectively. **B.** Group means at each time point are shown. The green and red plots identify hCSC-treated and vehicle control groups, respectively. The inset in panel B shows cumulative CK-MB levels. Data are mean±SEM. There were no significant differences in plasma CK-MB levels over the 1 month follow up between groups (P > 0.05 at each time point).

Myocardial injury resulting from possible micro-embolization and ischemia was assessed by serial measurements of plasma cTnI (Fig 6) and CK-MB levels (Fig 7). In both hCSC-treated and vehicle controls, cTnI levels rose slightly after catheterization and intracoronary infusion, peaking at 6 h post catheterization in both groups (hCSC treated, 1.3±0.68 vs. 0.08±0.08 ng/ml at baseline; controls, 1.4±0.58 vs, 0.07±0.08 ng/ml at baseline). In both groups, cTnI returned to baseline by 12 h and remained at baseline levels at 24 h, 1 week, and 1 month after infusion. At no time-point during the study was there a significant difference in plasma cTnI levels between the two groups. In addition, total cumulative myocardial cTnI release did not differ significantly between the hCSC-treated and vehicle-treated groups. Plasma levels of CK-MB exhibited a very slight but clinically irrelevant increase from baseline in both groups, (0.068±0.01 vs 0.039±0.01 ng/ml at baseline in the treated group; 0.058±0.013 vs 0.039±0.01 ng/ml at baseline in the control group) peaking at 12 h in the treatment group and 24 h in the control group. Importantly, neither peak mean nor cumulative enzyme levels differed significantly between vehicle-treated and hCSC-treated groups, indicating that hCSC delivery was not associated with myocardial injury(P>0.05).

### Intracoronary infusion of 20 million c-kit^pos^ hCSCs does not impair renal function

**Fig 8:**
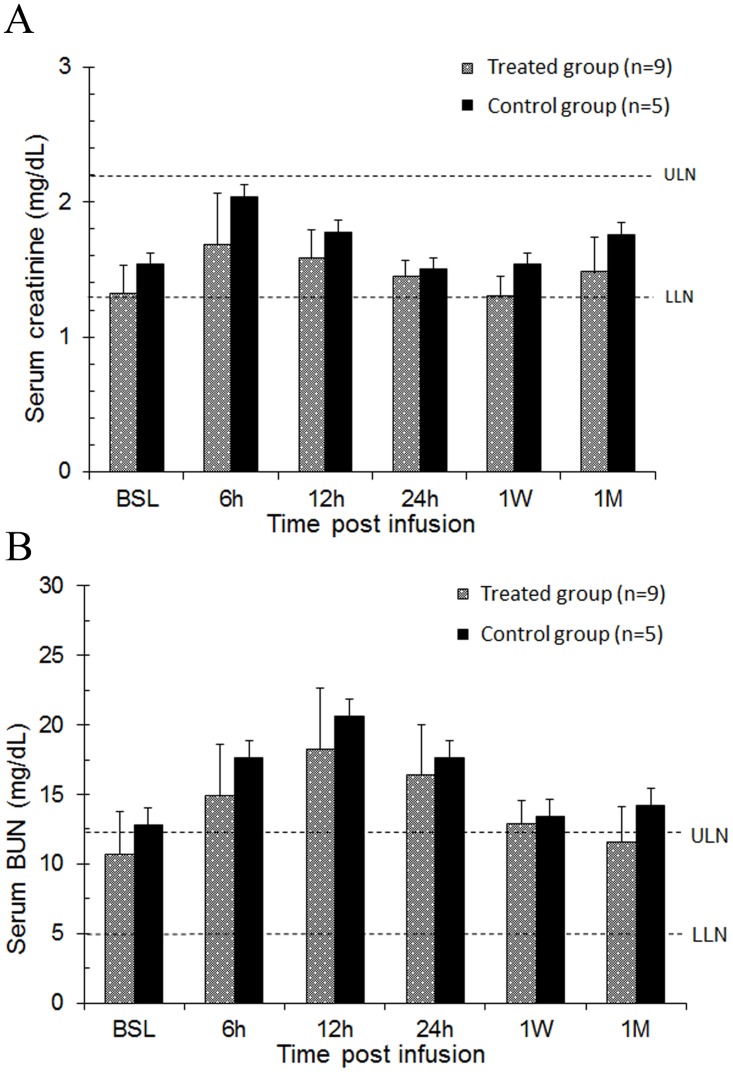
Intracoronary infusion of 20 million human c-kit^pos^ CSCs does not impair renal function. Renal function was assessed by serum creatinine and blood urea nitrogen (BUN) values over serial time-points (baseline, 6, 12, 24 h, 1 week, and 1 month). **A.** Bar graph of serum creatinine levels in hCSC-treated and vehicle control group. **B.** Bar graph of serum BUN levels in hCSC-treated and vehicle control group. Blue and purple bars identify hCSC-treated and vehicle control groups, respectively. Upper limits of normal (ULN) and lower limits of normal (LLN) in each graph are depicted by dashed lines respectively. Data are mean±SEM. There were no significant differences in serum creatinine or BUN levels over the 1 month follow up between groups (P > 0.05 at each time point).

Serum creatinine and BUN levels rose approximated 25% over baseline in the first 12–24 h in both the hCSC-treated and the vehicle groups (Fig 8). In the treated group, creatinine increased from 1.32 mg/dL to 1.68 mg/dL at 6 h after catheterization; a similar pattern was observed in the control group, in which creatinine increased from 1.54 mg/dL at baseline to 2.04 mg/dL at 6 h after catheterization. Serum creatinine levels returned to baseline values in both groups by 24 h, and no changes were observed at 1 week and 1 month (Fig 8A). Serum BUN levels showed a similar pattern of transient increase at 6 h followed by a return to baseline levels (Fig 8B). There was no significant difference at any time point between hCSC-treated and control pigs with respect to either creatinine or BUN serum levels, indicating that the slight increases observed at 6 h were not due to the infusion of hCSCs.

### Intracoronary infusion of 20 million c-kit^pos^ hCSCs does not impair hepatic function

**Fig 9:**
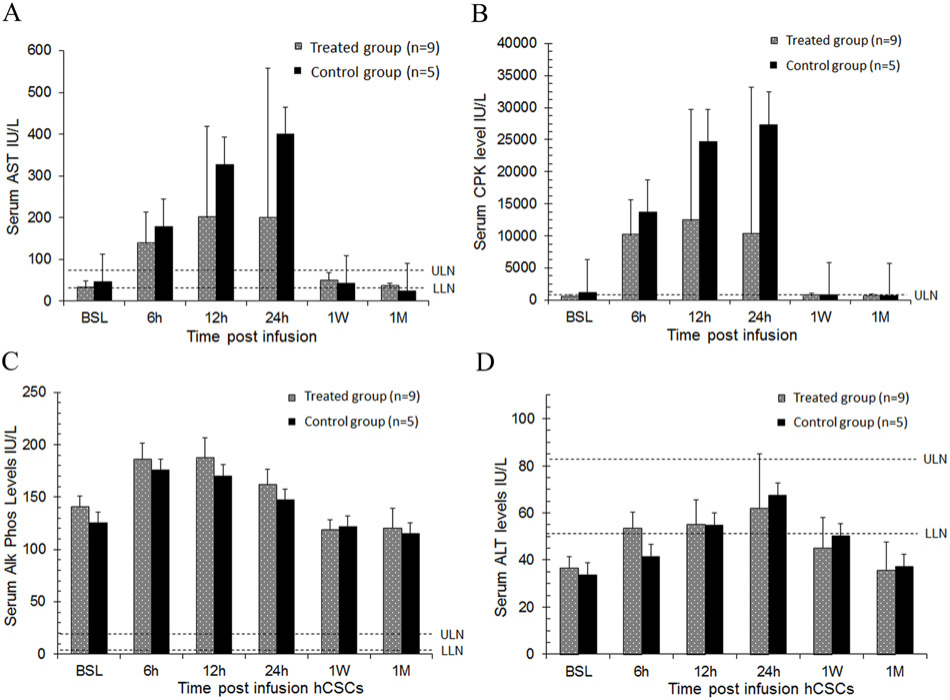
Intracoronary infusion of 20 million human c-kit^pos^ CSCs does not impair liver function. Liver function was assessed by serum AST, ALT, alkaline phosphatase, and total CK levels at serial time points (baseline, 6, 12, 24 h, 1 week, and 1 month). **A.** Serum aspartate aminotransferase (AST), **B.** Serum creatine phosphokinase (CPK), **C.** Serum alkaline phosphatase (Alk. Phos.), **D.** Serum alanine aminotransferase (ALT). Upper limits of normal) and lower limits of normal (LLN) in each graph are depicted by dashed lines respectively. Data are mean±SEM. There were no significant differences in serum AST, ALT, alkaline phosphatase, or total CK levels over the 1 month follow up between groups (P > 0.05 at each time point).

Serum AST, ALT, alkaline phosphatase, and total CPK levels were significantly higher after catheterization (P< 0.05 vs. baseline) in both hCSC-treated and control groups (Fig 9). Specifically, AST increased to levels four times (200–400 IU/L) the upper limit of normal (45–83 IU/L), peaking 24 h after catheterization (Fig 9A). ALT also increased but did not surpass the upper limits of normal (52–81 IU/L) in either treatment (61.8±23.6 IU/L) or control (67.6±23.0 IU/L) groups (Fig 9D). There was no significant difference between hCSC-treated and control pigs in either AST or ALT, indicating that the rise in these markers was not due to the infused cell product. All values were within normal limits 1 week and 1 month after catheterization (P>0.05 vs. baseline).

### Intracardiac retention of c-kit^pos^ hCSC in pigs 30 days post intracoronary infusion

**Fig 10:**
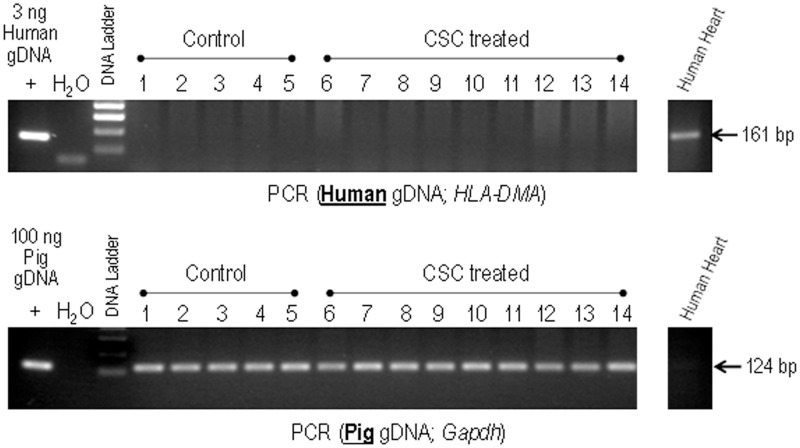
Detection of human CSCs in control versus hCSC-treated pig hearts. Genomic DNA isolated from representative LV sections from control (lanes 1–5) and human CSC-treated pigs (lanes 6–14) were analyzed by PCR for the presence of human genomic DNA (HLA-DMA). Samples were also analyzed for the presence of pig genomic DNA (Gapdh) as a control for DNA quality. Genomic DNA isolated from human heart sections was used as both positive and negative control. None of the samples, including CSC-treated ones, show detectable levels of human DNA.

To assess the number of hCSCs retained in the porcine heart 30 days after intracoronary infusion, genomic DNA was isolated from anterior portions of the left ventricle and analyzed by PCR for the presence of human genomic DNA (HLA-DMA) (Fig 10). No human DNA could be detected in any control (lanes 1–5) or human CSC-treated (lanes 6–14) LV samples, indicating the retention of human CSCs was minimal and/or below the detection limit of our assay (1 hCSC per 15, 000 porcine cells). Samples were also analyzed for the presence of pig genomic DNA (Gapdh) as a control for DNA quality. Porcine DNA was detected in all samples.

## Discussion

The present study was conducted to assess the safety of intracoronary infusion of 20 million c-kit^pos^ hCSCs. We have previously found in a porcine model of ischemic cardiomyopathy that intracoronary delivery of 500,000 c-kit^pos^ autologous CSCs, which is roughly equivalent to 1 million CSCs in humans, does not result in apparent cardiac injury [19]. The SCIPIO trial showed that intracoronary infusion of 1 million autologous c-kit^pos^ CSCs in humans is safe and may produce beneficial effects on cardiac function, myocardial scar size, and functional capacity [2]. However, the dose employed in SCIPIO was relatively low (compared with other trials that have infused, for example, 25 million [15] or even >200 million [21] cells). Evidence suggests that higher doses of cells may be more efficacious [3]. It is currently unknown whether doses of hCSCs greater than 1 million can be safely administered intracoronarily. Therefore, we conducted the present study with a dose of cells 40 times higher than that used in our previous study [19] as a preamble to future clinical trials. To our knowledge, our infused dose of 20 million human c-kit^pos^ CSCs delivered intracoronarily is the highest dose ever attempted in any clinical or preclinical model using hCSCs.

The results of the present study indicate that intracoronary infusion of 20 million c-kit^pos^ hCSCs is safe. This higher dose did not result in any evidence of myocardial injury, impairment in regional or global cardiac function, deterioration of renal or hepatic function, or other adverse effects, either in the immediate post-infusion period or during the subsequent month, compared with vehicle (Figs 5–9). Very small, transient increases in plasma troponin I were observed after catheterization in both groups (Fig 6) but were not associated with any measurable impairment in LV function (Fig 5). These low-level, transient increases in plasma troponin are not dissimilar from those seen in prior studies[19]; given their low magnitude and brief duration, they are likely insignificant. Importantly, since they were observed in both groups, they cannot be attributed to the hCSC product. We suspect that these early transient increases in plasma troponin I in both hCSC- and vehicle-treated animals may have been, at least in part, a result of the observed impairment in renal function caused by exposure to cyclosporine and a large intravenous contrast load. Indeed, the time-course of renal dysfunction coincided with the transient rise and resolution of plasma troponin over the first 12–24 h following catheterization. Renal dysfunction, decreased creatinine clearance, and uremia have been implicated in increased plasma levels of troponin in patients without acute myocardial injury [22]. In clinical studies in which elevations of plasma troponin were related to renal dysfunction, acute myocardial injury was excluded by observing a lack of parallel elevations in plasma CK-MB [22]. This pattern of low-level troponin increase with minimal or no concurrent increase in CK-MB during a period of renal impairment mirrors the pattern of cardiac biomarkers observed in the present study. It is possible that other factors associated with cardiac catheterization also contributed to the slight rise in plasma troponin. In any case, the fact that vehicle-treated pigs exhibited similar elevations despite receiving no hCSCs indicates the elevations in treated pigs were not caused by hCSCs but, rather, by mechanisms that affected both groups.

Our findings are consistent with previous studies documenting the safety of intracoronary infusion (without stop flow) of similar numbers of other cell types, including bone marrow derived MSCs[23,24]. Specifically, Suzuki et al[23] demonstrated the safety and efficacy of 45 million MSCs infused intracoronarily without stop-flow; the authors observed myocyte regeneration and improved cardiac function without any evidence of myocardial necrosis related to the infusion [23]. The same group observed a similar safety and efficacy profile following intracoronary infusion (without stop flow) of 30 million cardiosphere-derived cells divided among each of three coronary vessels[24]. Again, no significant TnI elevation was observed.

As mentioned above, after the catheterization procedure renal function was transiently impaired in both treated and control groups (Fig 8), indicating that the decline in creatinine clearance was not due to systemic distribution of hCSCs and renal microembolization. The mild degree and brief duration of renal impairment, along with the complete return of creatinine and BUN to baseline levels within 24 h after catheterization in both groups, indicates that the etiology was most likely related to the contrast administered intravenously during coronary angiography. Contrast-induced nephropathy (CIN) is commonly observed in clinical settings, with temporary decreases in creatinine clearance that usually return to normal values within 24–48 h [25]. Concomitant exposure of pigs to cyclosporine likely predisposed to the occurrence of CIN and decreased creatinine clearance, since both cyclosporine and intravenous contrast exposure produce vasoconstriction of renal afferent arterioles [25,26], which in turn decreases the glomerular filtration rate.

A slight, transient impairment in liver function was also observed (Fig 9). But again, this phenomenon was seen in both hCSC-treated and vehicle-treated pigs, and the duration and magnitude were not significantly different between groups, indicating that the elevated levels of AST, ALT, and alkaline phosphatase were not caused by intracoronarily delivered hCSCs. ALT, being more specific for liver injury than either AST or alkaline phosphatase [27], did not increase above the upper limits of normal (51–82 IU/L) (Fig 9D). In fact, a hepatic source of these elevated biomarkers is highly unlikely. While leak of aminotransferases into the systemic circulation can indicate liver injury, the liver is not the only source of these biomarkers and caution should be observed in clinical interpretation [27]. Increases in these biomarkers are also seen with skeletal muscle injury [27,28]. In our porcine model, a cut-down procedure was used to provide unrestricted access to the femoral artery for arterial sheath and catheter placement. The resultant soft tissue and skeletal muscle injury provides a non-hepatic source of elevated transaminases. This is confirmed by the concurrent increase in muscle specific creatine phosphokinase (CPK) (Fig 9B), which mirrored the magnitude and duration of the rise in transaminase levels (Fig 9A). All of these markers returned to baseline levels concurrently.

Despite the high dose of 20 million cells, myocardial retention of c-kit^pos^ hCSCs 30 days after intracoronary infusion was observed to be minimal and below the threshold of detection as measured by PCR targeting human genomic DNA. In our experience, this finding is neither surprising nor unexpected. Previous studies [29–31] have also found minimal retention 30–35 days after c-kit^pos^ CSC administration in rodent models. Additionally, in the current study, human c-kit^pos^ hCSC were infused as a xenograft, and only cyclosporine was utilized to prevent acute rejection. The animal’s immune reaction to these human cells is not likely to be completely suppressed by cyclosporine alone over a 35-day time period, and thus is likely to have contributed to the attrition of the human cells via immune clearance. In a recent study performed to quantify hCSC engraftment and retention in pigs after intracoronary infusion of 10 million Indium^111^-radiolabeled c-kit^pos^ hCSCs, we found that just 4–5% of the infused cells remained in the entire heart at 24 h [32]. Additionally, in this study we compared intracoronary infusion both with and without utilization of the stop-flow technique, and found no significant difference between the two methodologies with respect to hCSC retention at 24 h [32]. This would suggest that the negligible level of hCSC cardiac retention observed in the present study was not due to the fact that we did not use the stop flow technique. However, it should be pointed out that in the current study we infused cells into normal, noninfarcted hearts (this was done in order to maximize the sensitivity of our model in detecting any myocardial damage caused by the high dose of 20 million hCSCs). This may have accelerated the disappearance of hCSCs from the myocardium by negatively impacting the homing and/or adhesion of the hCSCs to damaged tissue that occurs in the setting of ischemic cardiomyopathy [33,34].

In conclusion, intracoronary infusion of 20 million human c-kit^pos^ CSCs in pigs (equivalent to ~40 million hCSCs in humans) does not cause acute myocardial injury, impaired regional or global myocardial function, adverse changes in LV dimensions, or liver and renal injury. These results have immediate translational value and lay the groundwork for using doses of CSCs >1 million in future clinical trials. Further studies are needed to determine whether doses of c-kit^pos^ hCSCs >1 million result in greater efficacy in patients with ischemic cardiomyopathy.

## Supporting Information

S1 Table c-kit positivity and cell product viability dataset. (Reference Fig 4).
(PDF)

S2 Table Echocardiographic dataset. (Reference Fig 5).
(PDF)

S3 Table Troponin I dataset. (Reference Fig 6).
(PDF)

S4 Table CK-MB dataset. (Reference Fig 7).
(PDF)

S5 Table Creatinine dataset. (Reference Fig 8A).
(PDF)

S6 Table BUN dataset. (Reference Fig 8B).
(PDF)

S7 Table AST dataset. (Reference Fig 9A).
(PDF)

S8 Table Total CPK dataset. (Reference Fig 9B).
(PDF)

S9 Table Alk Phos dataset. (Reference Fig 9C).
(PDF)

S10 Table ALT dataset. (Reference Fig 9D).
(PDF)
