# Supplementary material for: Safety of Intracoronary Infusion of 20 Million C-Kit Positive Human Cardiac Stem Cells in Pigs
Source: PLoS One. 2015 Apr 23;10(4):e0124227. doi: 10.1371/journal.pone.0124227 (PMC4408046; doi:10.1371/journal.pone.0124227)
Supplement: S2 Table — (Reference Fig 5). (PDF) [file pone.0124227.s002.pdf]

S2 Table: Echocardiographic data. (Reference Fig. 5)

Pig#	Group	time point	Anterior Wall Thickness at diastole (cm)	LV End-Diastolic Diameter (cm)	Posterior Wall Thickness at diastole (cm)	Anterior Wall Thickness at systole (cm)	LV End-Systolic Diameter (cm)	Posterior Wall Thickness at systole (cm)	LV End-Diastolic Area (cm^2)	LV End-Systolic Area (cm^2)	Fractional Shortening (%)	Fractional Area Change (%)	Ejection Fraction (%, M-mode)	Ejection Fraction (%, volumetric)	LV End-Diastolic Volume (mL, Teicholz)	LV End-Systolic Volume (mL, Teicholz)	Anterior Wall Thickening Fraction (%)	Posterior Wall Thickening Fraction (%)
Pig#91083	Vehicle	BSL	0.71	3.85	0.92	1.13	2.80	1.21	10.82	6.14	27.27	43.25	47.11	53.77	63.91	29.55	59.15	31.52
Pig#90960	Vehicle	BSL	0.76	3.60	0.67	1.05	2.46	1.03	8.76	4.46	31.67	49.09	53.31	60.61	54.43	21.44	38.71	54.42
Pig#90961	Vehicle	BSL	0.65	3.93	0.90	1.26	2.36	1.29	13.08	5.94	39.95	54.59	63.94	71.20	67.12	19.33	93.85	43.33
Pig#90963	Vehicle	BSL	0.71	3.36	0.81	1.15	2.36	1.13	7.83	4.48	29.76	42.78	50.67	58.07	46.10	19.33	61.97	39.51
Pig#90964	Vehicle	BSL	0.64	4.36	0.80	1.17	3.04	1.35	16.88	8.75	30.28	48.16	51.38	57.88	85.82	36.15	82.81	68.75
		mean	0.69	3.82	0.82	1.15	2.60	1.20	11.47	5.95	31.79	47.57	53.28	60.30	63.48	25.16	67.30	47.51
		SEM	0.02	0.17	0.04	0.03	0.14	0.06	1.63	0.78	2.16	2.16	2.85	2.94	6.70	3.33	9.64	6.47
		n	5.00	5.00	5.00	5.00	5.00	5.00	5.00	5.00	5.00	5.00	5.00	5.00	5.00	5.00	5.00	5.00
Pig#91083	Vehicle	24h	0.66	3.82	0.86	1.05	2.64	1.24	14.24	6.49	30.89	54.42	52.24	59.26	62.73	25.56	59.09	44.19
Pig#90960	Vehicle	24h	0.71	3.86	0.60	1.13	2.77	0.73	12.49	6.49	28.24	48.04	48.50	55.25	64.31	28.78	59.15	21.67
Pig#90961	Vehicle	24h	0.62	3.86	0.80	1.23	2.51	1.25	13.86	5.25	34.97	62.12	57.72	64.94	64.31	22.54	98.39	56.25
Pig#90963	Vehicle	24h	0.70	3.19	0.69	1.07	2.25	1.00	11.50	7.22	29.47	37.22	50.25	57.82	40.65	17.15	53.08	45.77
Pig#90964	Vehicle	24h	0.66	4.27	0.83	1.11	2.96	0.98	13.97	6.83	30.68	51.11	51.95	58.55	81.71	33.87	68.18	18.07
		mean	0.67	3.80	0.76	1.12	2.63	1.04	13.21	6.46	30.85	50.58	52.13	59.17	62.74	25.58	67.58	37.19
		SEM	0.02	0.17	0.05	0.03	0.12	0.10	0.52	0.33	1.14	4.08	1.55	1.60	6.53	2.82	8.07	7.39
		n	5.00	5.00	5.00	5.00	5.00	5.00	5.00	5.00	5.00	5.00	5.00	5.00	5.00	5.00	5.00	5.00
Pig#91083	Vehicle	1Wk	0.68	4.06	0.81	1.16	2.79	1.11	12.07	4.44	31.28	63.21	52.78	59.61	72.52	29.29	70.59	37.04
Pig#90960	Vehicle	1Wk	0.68	3.73	0.71	1.16	2.66	1.05	12.60	6.86	28.69	45.56	49.14	56.06	59.26	26.04	70.59	47.89
Pig#90961	Vehicle	1Wk	0.66	4.09	0.80	1.37	2.71	1.33	11.35	5.10	33.74	55.07	56.10	63.05	73.79	27.26	107.58	66.25
Pig#90963	Vehicle	1Wk	0.67	3.29	0.78	1.06	2.40	1.17	9.97	5.57	27.05	44.13	46.79	53.98	43.81	20.16	58.21	50.00
Pig#90964	Vehicle	1Wk	0.61	4.29	0.82	1.14	3.01	1.21	11.64	7.07	29.84	39.26	50.77	57.29	82.61	35.29	86.89	47.56
		mean	0.66	3.89	0.78	1.18	2.71	1.17	11.53	5.81	30.12	49.45	51.11	58.00	66.40	27.61	78.77	49.75
		SEM	0.01	0.18	0.02	0.05	0.10	0.05	0.44	0.51	1.14	4.29	1.59	1.56	6.77	2.45	8.52	4.70
		n	5.00	5.00	5.00	5.00	5.00	5.00	5.00	5.00	5.00	5.00	5.00	5.00	5.00	5.00	5.00	5.00
Pig#91083	Vehicle	1Mo	0.68	4.23	0.88	1.21	2.93	1.33	12.98	6.88	30.73	47.00	52.02	58.66	79.91	33.03	77.94	51.14
Pig#90960	Vehicle	1Mo	0.69	3.86	0.71	1.00	2.73	1.22	12.45	6.89	29.27	44.66	49.98	56.83	64.31	27.76	44.93	71.83
Pig#90961	Vehicle	1Mo	0.71	4.16	0.89	1.23	2.88	1.33	13.21	6.52	30.77	50.64	52.07	58.77	76.82	31.67	73.24	49.44
Pig#90963	Vehicle	1Mo	0.62	3.80	0.81	1.11	2.53	1.28	13.12	7.09	33.42	45.96	55.67	62.88	61.95	22.99	79.03	58.02
Pig#90964	Vehicle	1Mo	0.67	4.56	0.91	1.18	3.13	1.33	14.06	7.58	31.36	46.09	52.89	59.30	95.36	38.82	76.12	46.15
		mean	0.67	4.12	0.84	1.15	2.84	1.30	13.16	6.99	31.11	46.87	52.53	59.29	75.67	30.86	70.25	55.32
		SEM	0.02	0.14	0.04	0.04	0.10	0.02	0.26	0.17	0.67	1.01	0.92	0.99	6.02	2.65	6.41	4.56
		n	5.00	5.00	5.00	5.00	5.00	5.00	5.00	5.00	5.00	5.00	5.00	5.00	5.00	5.00	5.00	5.00
Pig#91079	CSCs	BSL	0.61	3.27	0.62	0.99	2.47	0.87	10.83	7.50	24.46	30.75	42.94	49.82	43.17	21.66	62.95	40.10
Pig#91080	CSCs	BSL	0.74	3.81	0.81	1.11	2.64	1.20	11.19	5.96	30.71	46.74	51.99	59.01	62.34	25.56	50.00	48.15
Pig#91081	CSCs	BSL	0.65	3.70	0.60	1.02	2.50	0.97	13.20	7.32	32.43	44.55	54.35	61.60	58.13	22.32	57.41	61.81
Pig#91082	CSCs	BSL	0.74	3.97	0.68	1.12	2.99	1.13	10.09	5.90	24.69	41.53	43.28	49.51	68.76	34.72	51.35	66.18
Pig#91086	CSCs	BSL	0.69	4.29	0.73	1.13	3.06	1.08	12.66	6.52	28.67	48.50	49.12	55.53	82.61	36.73	63.77	47.95
Pig#91084	CSCs	BSL	0.57	3.61	0.61	1.06	2.32	0.99	10.36	5.03	35.73	51.45	58.70	66.20	54.80	18.52	85.96	62.30
Pig#91085	CSCs	BSL	0.80	4.35	0.75	1.06	3.15	1.20	14.08	9.16	27.59	34.94	47.56	53.82	85.36	39.42	32.50	60.00
Pig#90959	CSCs	BSL	0.69	3.71	0.77	1.17	2.70	0.98	9.24	4.73	27.22	48.81	47.04	53.82	58.50	27.02	69.57	27.27
Pig#90962	CSCs	BSL	0.69	3.69	0.70	1.05	2.71	1.01	10.46	6.68	26.56	36.14	46.06	52.79	57.75	27.26	52.39	44.08
		mean	0.69	3.82	0.70	1.08	2.73	1.05	11.35	6.53	28.67	42.60	49.00	55.79	63.49	28.13	58.43	50.87
		SEM	0.02	0.11	0.03	0.02	0.10	0.04	0.54	0.45	1.23	2.40	1.73	1.84	4.48	2.42	4.95	4.25
		n	9.00	9.00	9.00	9.00	9.00	9.00	9.00	9.00	9.00	9.00	9.00	9.00	9.00	9.00	9.00	9.00
Pig#91079	CSCs	24h	0.61	3.79	0.66	0.82	2.79	1.00	11.33	6.32	26.39	44.22	45.81	52.42	61.56	29.29	34.43	51.52
Pig#91080	CSCs	24h	0.68	3.84	0.82	1.07	2.68	1.20	11.00	5.44	30.21	50.55	51.29	58.24	63.52	26.52	57.35	46.34
Pig#91081	CSCs	24h	0.67	3.61	0.73	0.99	2.40	0.96	8.98	4.40	33.52	51.00	55.80	63.21	54.80	20.16	47.76	31.51
Pig#91082	CSCs	24h	0.72	4.30	0.67	1.18	3.10	1.02	11.08	5.49	27.91	50.45	48.03	54.36	83.07	37.92	63.89	52.24
Pig#91086	CSCs	24h	0.68	4.12	0.71	0.95	3.00	1.02	10.66	5.16	27.18	51.59	46.98	53.38	75.08	35.00	39.15	43.26
Pig#91084	CSCs	24h	0.62	3.89	0.78	1.12	2.73	1.06	9.95	4.60	29.82	53.77	50.75	57.62	65.51	27.76	80.65	35.90
Pig#91085	CSCs	24h	0.75	3.85	0.85	1.14	2.56	1.25	11.23	5.64	33.51	49.78	55.79	62.95	63.91	23.68	52.00	47.06
Pig#90959	CSCs	24h	0.84	3.79	0.82	1.27	2.60	1.10	10.86	5.49	31.40	49.45	52.94	60.03	61.56	24.61	51.19	34.15
Pig#90962	CSCs	24h	0.67	4.35	0.61	1.28	3.26	0.89	16.10	8.25	25.06	48.76	43.84	49.80	85.36	42.85	91.04	45.90
		mean	0.69	3.95	0.74	1.09	2.79	1.06	11.24	5.64	29.44	49.95	50.13	56.89	68.26	29.75	57.50	43.10
		SEM	0.02	0.08	0.03	0.05	0.09	0.04	0.66	0.38	1.01	0.86	1.43	1.57	3.49	2.46	6.16	2.51
		n	9.00	9.00	9.00	9.00	9.00	9.00	9.00	9.00	9.00	9.00	9.00	9.00	9.00	9.00	9.00	9.00
Pig#91079	CSCs	1Wk	0.65	4.10	0.81	1.14	2.85	1.10	12.80	6.65	30.49	48.05	51.68	58.41	74.22	30.87	75.38	35.80
Pig#91080	CSCs	1Wk	0.70	3.79	0.78	1.06	2.81	1.11	12.13	8.61	25.86	29.02	45.03	51.58	61.56	29.81	51.43	42.31
Pig#91081	CSCs	1Wk	0.58	3.52	0.65	0.96	2.42	0.97	11.27	5.59	31.25	50.40	52.73	60.09	51.57	20.58	65.52	49.23
Pig#91082	CSCs	1Wk	0.72	4.33	0.61	1.20	3.07	0.95	16.86	8.65	29.10	48.70	49.73	56.15	84.44	37.03	66.67	55.74
Pig#91086	CSCs	1Wk	0.70	3.92	0.71	1.23	2.55	1.17	11.35	4.43	34.95	60.97	57.68	64.85	66.72	23.45	75.71	64.79
Pig#91084	CSCs	1Wk	0.76	3.67	0.72	1.27	2.34	1.19	10.52	4.13	36.24	60.74	59.35	66.81	57.00	18.92	67.11	65.28
Pig#91085	CSCs	1Wk	0.76	3.78	0.85	1.18	2.74	1.16	11.21	5.78	27.51	48.44	47.46	54.21	61.18	28.01	55.26	36.47
Pig#91059	CSCs	1Wk	0.76	3.78	0.85	1.18	2.74	1.16	11.67	6.30	27.51	46.02	47.46	54.21	61.18	28.01	55.26	36.47
Pig#90962	CSCs	1Wk	0.76	3.77	0.72	1.34	2.66	1.02	11.20	5.46	29.44	51.25	50.22	57.17	60.79	26.04	76.32	41.67
		mean	0.71	3.85	0.74	1.17	2.69	1.09	12.11	6.18	30.26	49.29	51.26	58.16	64.30	26.97	65.41	47.53
		SEM	0.02	0.08	0.03	0.04	0.08	0.03	0.63	0.53	1.15	3.11	1.58	1.68	3.26	1.85	3.19	3.96
		n	9.00	9.00	9.00	9.00	9.00	9.00	9.00	9.00	9.00	9.00	9.00	9.00	9.00	9.00	9.00	9.00
Pig#91079	CSCs	1Mo	0.66	4.64	0.63	1.37	3.35	0.98	19.22	10.04	27.80	47.76	47.87	53.92	99.33	45.77	107.58	55.56
Pig#91080	CSCs	1Mo	0.71	4.18	0.76	1.29	2.99	1.07	15.71	9.00	28.47	42.71	48.83	55.32	77.70	34.72	81.69	40.79
Pig#91081	CSCs	1Mo	0.64	3.55	0.66	1.11	2.43	0.92	10.88	6.09	31.55	44.03	53.15	60.49	52.63	20.80	73.44	39.39
Pig#91082	CSCs	1Mo	0.66	4.55	0.69	1.04	3.37	0.99	16.43	9.40	25.93	42.79	45.14	51.06	94.87	46.43	57.34	43.73
Pig#91086	CSCs	1Mo	0.70	3.54	0.81	1.18	2.23	1.20	11.25	5.91	37.01	47.47	60.32	67.93	52.28	16.77	68.57	48.15
Pig#91084	CSCs	1Mo	0.67	3.80	0.70	1.44	2.44	1.03	11.10	5.08	35.79	54.23	58.77	66.09	61.95	21.01	114.93	47.14
Pig#91085	CSCs	1Mo	0.66	3.92	0.76	1.09	2.88	1.15	11.49	7.13	26.53	37.95	46.02	52.53	66.72	31.67	65.15	51.32
Pig#91059	CSCs	1Mo	0.65	4.22	0.76	1.20	2.92	1.12	12.09	6.86	30.81	43.26	52.12	58.78	79.47	32.76	84.62	47.37
Pig#90962	CSCs	1Mo	0.66	3.89	0.74	1.14	2.78	1.22	12.54	6.04	28.53	51.83	48.93	55.68	65.51	29.03	72.73	64.86
		mean	0.67	4.03	0.72	1.21	2.82	1.08	13.41	7.28	30.27	45.78	51.24	57.98	72.27	30.99	80.67	48.70
		SEM	0.01	0.13	0.02	0.04	0.13	0.03	0.99	0.59	1.31	1.68	1.79	1.97	5.64	3.50	6.41	2.62
		n	9.00	9.00	9.00	9.00	9.00	9.00	9.00	9.00	9.00	9.00	9.00	9.00	9.00	9.00	9.00	9.00
		Vehicle	0.69	3.82	0.82	1.15	2.60	1.20	11.47	5.95	31.79	47.57	53.28	60.30	63.48	25.16	67.30	47.51
		(n=5)	0.02	0.17	0.04	0.03	0.14	0.06	1.63	0.78	2.16	2.16	2.85	2.94	6.70	3.33	9.64	6.47
		CSCs	0.69	3.82	0.70	1.08	2.73	1.05	11.35	6.53	28.67	42.60	49.00	55.79	63.49	28.13	58.43	50.87
		(n=9)	0.02	0.11	0.03	0.02	0.10	0.04	0.54	0.45	1.23	2.40	1.73	1.84	4.48	2.42	4.95	4.25
		P=	0.85	0.99	0.02	0.06	0.47	0.04	0.93	0.50	0.20	0.20	0.20	0.19	1.00	0.48	0.38	0.66
		Vehicle	0.67	3.80	0.76	1.12	2.63	1.04	13.21	6.46	30.85	50.58	52.13	59.17	62.74	25.58	67.58	37.19
		(n=5)	0.02	0.17	0.05	0.03	0.12	0.10	0.52	0.33	1.14	4.08	1.55	1.60	6.53	2.82	8.07	7.39
		CSCs	0.69	3.95	0.74	1.09	2.79	1.06	11.24	5.64	29.44	49.95	50.13	56.89	68.26	29.75	57.50	43.10
		(n=9)	0.02	0.08	0.03	0.05	0.09	0.04	0.66	0.38	1.01	0.86	1.43	1.57	3.49	2.46	6.16	2.51
		P=	0.50	0.40	0.76	0.72	0.30	0.86	0.07	0.18	0.40	0.85	0.39	0.37	0.43	0.31	0.34	0.37
		Vehicle	0.66	3.89	0.78	1.18	2.71	1.17	11.53	5.81	30.12	49.45	51.11	58.00	66.40	27.61	78.77	49.75
		(n=5)	0.01	0.18	0.02	0.05	0.10	0.05	0.44	0.51	1.14	4.29	1.59	1.56	6.77	2.45	8.52	4.70
		CSCs	0.71	3.85	0.74	1.17	2.69	1.09	12.11	6.18	30.26	49.29	51.26	58.16	64.30	26.97	65.41	47.53
		(n=9)	0.02	0.08	0.03	0.04	0.08	0.03	0.63	0.53	1.15	3.11	1.58	1.68	3.26	1.85	3.19	3.96
		P=	0.12	0.81	0.35	0.94	0.83	0.15	0.54	0.66	0.94	0.98	0.95	0.95	0.76	0.84	0.10	0.73
		Vehicle	0.67	4.12	0.84	1.15	2.84	1.30	13.16	6.99	31.11	46.87	52.53	59.29	75.67	30.86	70.25	55.32
		(n=5)	0.02	0.14	0.04	0.04	0.10	0.02	0.26	0.17	0.67	1.01	0.92	0.99	6.02	2.65	6.41	4.56
		CSCs	0.67	4.03	0.72	1.21	2.82	1.08	13.41	7.28	30.27	45.78	51.24	57.98	72.27	30.99	80.67	48.70
		(n=9)	0.01	0.13	0.02	0.04	0.13	0.03	0.99	0.59	1.31	1.68	1.79	1.97	5.64	3.50	6.41	2.62
		P=	0.69	0.67	0.01	0.39	0.92	0.00	0.86	0.73	0.66	0.66	0.62	0.65	0.71	0.98	0.31	0.20
