# Supplementary material for: Safety of Intracoronary Infusion of 20 Million C-Kit Positive Human Cardiac Stem Cells in Pigs
Source: PLoS One. 2015 Apr 23;10(4):e0124227. doi: 10.1371/journal.pone.0124227 (PMC4408046; doi:10.1371/journal.pone.0124227)
Supplement: S3 Table — (Reference Fig 6). (PDF) [file pone.0124227.s003.pdf]

S3 Table: Troponin I. (Reference Fig. 6)

	Plasma cTnI (ng/ml) in Pigs with hCSC Intracoronary Administration							
Pig#	Group Assignment	BSL	6 h	12 h	24 h	1wk	30d	Cumulative cTnI
91083	Vehicle	0	0.282	0.06	0.034	0.01	0	0.3485
90960	Vehicle	0	3.56	2.396	1.935	0	0	8.07725
90961	Vehicle	0.374	1.62	0.871	0.337	0	0	2.247625
90963	Vehicle	0	0.708	2.353	0.737	0	0.02	3.684625
90964	Vehicle	0	0.933	0.394	0.112	0.021	0	1.0495
Mean		0.0748	1.4206	1.2148	0.631	0.0062	0.004	3.0815
SEM		0.0748	0.577008	0.490724	0.348128	0.004176	0.004	1.371403266
n		5	5	5	5	5	5	5
91079	CSCs	0	2.001	1.271	0.343	0	0.02	2.321625
91080	CSCs	0.676	6.459	4.638	2.709	0.591	0.144	23.14425
91081	CSCs	0	0	0.845	0.259	0	0	1.158625
91082	CSCs	0	0	0	0	0	0	0
91084	CSCs	0	0	0.077	0	0	0	0.028875
91085	CSCs	0.007	0.743	0.224	0.113	0.012	0	0.818875
91086	CSCs	0.038	0.794	0.201	0.13	0	0	0.739125
90959	CSCs	0	0.754	0.459	0.027	0	0.006	0.517375
90962	CSCs	0.018	1.149	0.307	0.254	0.03	0.003	1.717625
Mean		0.082111	1.322222	0.891333	0.426111	0.070333	0.019222	3.382930556
SEM		0.07436	0.677613	0.487221	0.288295	0.065171	0.015748	2.482791604
n		9	9	9	9	9	9	9
P vs. Vehicle		0.950351	0.924369	0.675381	0.668576	0.485397	0.496527	0.933414336
