# Supplementary material for: Safety of Intracoronary Infusion of 20 Million C-Kit Positive Human Cardiac Stem Cells in Pigs
Source: PLoS One. 2015 Apr 23;10(4):e0124227. doi: 10.1371/journal.pone.0124227 (PMC4408046; doi:10.1371/journal.pone.0124227)
Supplement: S4 Table — (Reference Fig 7). (PDF) [file pone.0124227.s004.pdf]

S4 Table: CK-MB. (Reference Fig. 7)

	Plasma CK-MB (ng/ml) in Pigs with hCSC Intracoronary Administration							
Pig#	Group Assignment	BSL	6 h	12 h	24 h	1wk	30d	Cumulative cTnI
91083	Vehicle	0.046	0.038	0.039	0.04	0.021	0.077	1.395875
90960	Vehicle	0.027	0.017	0.037	0.077	0.036	0.052	1.41875
90961	Vehicle	0.059	0.062	0.044	0.095	0.057	0.061	1.935125
90963	Vehicle	0.046	0.047	0.016	0.037	0.082	0.044	1.88475
90964	Vehicle	0.015	0.03	0.041	0.028	0.075	0.049	1.78175
Mean		0.0386	0.0388	0.0354	0.0554	0.0542	0.0566	1.68325
SEM		0.007801	0.007612	0.004986	0.012964	0.011504	0.005802	0.11538
n		5	5	5	5	5	5	5
91079	CSCs	0.052	0.04	0.096	0.033	0.021	0.061	1.21775
91080	CSCs	0.028	0.018	0.037	0.029	0.024	0.046	1.021125
91081	CSCs	0.012	0.048	0.058	0.063	0.003	0.047	0.836
91082	CSCs	0.022	0.049	0.034	0.032	0	0.055	0.78625
91084	CSCs	0	0.072	0.064	0.075	0.014	0.068	1.27075
91085	CSCs	0.021	0.046	0.129	0.099	0.047	0.056	1.73075
91086	CSCs	0.089	0.059	0.087	0.059	0.051	0.048	1.63075
90959	CSCs	0.066	0.059	0.049	0.04	0.046	0.068	1.686375
90962	CSCs	0.062	0.075	0.055	0.056	0.068	0.058	1.944125
Mean		0.039111	0.051778	0.067667	0.054	0.030444	0.056333	1.34709
SEM		0.009797	0.005759	0.010293	0.007748	0.007844	0.002794	0.13963
n		9	9	9	9	9	9	9
2-way RM ANOVA	P vs. Vehicle	0.189	0.577	0.068	0.448	0.002 (ctrl higher)	0.919	0.369
