# Supplementary material for: Safety of Intracoronary Infusion of 20 Million C-Kit Positive Human Cardiac Stem Cells in Pigs
Source: PLoS One. 2015 Apr 23;10(4):e0124227. doi: 10.1371/journal.pone.0124227 (PMC4408046; doi:10.1371/journal.pone.0124227)
Supplement: S5 Table — (Reference Fig 8A). (PDF) [file pone.0124227.s005.pdf]

S5 Table: Creatinine. (Reference Fig. 8A)

**Creatinine (mg/dl) dataset**						
Treatment (Tx)						
	BSL	6h	12h	24h	1wk	1mo
91079	1.2	1.7	1.6	1.3	1.1	1.1
91080	1.1	1.5	1.4	1.2	1.2	1.6
91081	1.3	1.7	1.8	1.4	1.4	1.6
91082	1.4	1.8	1.5	1.3	1.2	1.3
91084	1.3	1.4	1.4	1.5	1.3	1.5
91085	1.2	1.5	1.4	1.3	1.2	1.3
91086	1.8	2	1.9	1.8	1.6	1.9
90959	1.2	1.8	1.6	1.5	1.3	1.6
90962	1.4	1.7	1.6	1.7	1.4	1.4
Average Tx Group (n=9)	1.322222	1.677778	1.577778	1.444444	1.3	1.477778
Std Deviation Tx Group	0.204803	0.185592	0.17873	0.200693	0.15	0.233333
Control (Ctrl)						
	BSL	6h	12h	24h	1W	1M
(Ctrl) 91083	1.4	1.6	1.6	1.6	1.5	1.6
(Ctrl) 90960	1.8	2.5	1.9	1.5	1.6	1.8
(Ctrl) 90961	1.5	2.1	1.9	1.6	1.7	2
(Ctrl) 90963	1.7	2.3	2	1.5	1.6	2
(Ctrl) 90964	1.3	1.7	1.5	1.3	1.3	1.4
Average Control Group (n=5)	1.54	2.04	1.78	1.5	1.54	1.76
Std Deviation Control Group	0.207364	0.384708	0.216795	0.122474	0.151658	0.260768
