# Supplementary material for: Safety of Intracoronary Infusion of 20 Million C-Kit Positive Human Cardiac Stem Cells in Pigs
Source: PLoS One. 2015 Apr 23;10(4):e0124227. doi: 10.1371/journal.pone.0124227 (PMC4408046; doi:10.1371/journal.pone.0124227)
Supplement: S6 Table — (Reference Fig 8B). (PDF) [file pone.0124227.s006.pdf]

S6 Table: BUN. (Reference Fig. 8B)

**BUN (mg/dl) dataset**						
Treatment (Tx)						
	BSL	6h	12h	24h	1wk	1mo
91079	11	16	25	19	10	8
91080	8	13	14	12	13	13
91081	14	19	24	20	13	14
91082	10	14	16	14	12	10
91084	11	15	18	18	16	14
91085	11	16	18	17	14	11
91086	13	14	17	15	13	12
90959	8	12	17	19	12	13
90962	10	15	15	14	13	9
Average Tx Group (n=9)	10.66667	14.88889	18.22222	16.44444	12.88889	11.55556
Std Deviation Tx Group	2	2.027588	3.800585	2.788867	1.615893	2.185813
Control (Ctrl)						
	BSL	6h	12h	24h	1W	1M
(Ctrl) 91083	12	13	15	14	12	10
(Ctrl) 90960	16	23	25	23	13	15
(Ctrl) 90961	15	17	20	15	14	15
(Ctrl) 90963	13	19	25	19	16	17
(Ctrl) 90964	8	16	18	17	12	14
Average Control Group (n=5)	12.8	17.6	20.6	17.6	13.4	14.2
Std Deviation Control Group	3.114482	3.714835	4.393177	3.577709	1.67332	2.588436
