# Supplementary material for: Safety of Intracoronary Infusion of 20 Million C-Kit Positive Human Cardiac Stem Cells in Pigs
Source: PLoS One. 2015 Apr 23;10(4):e0124227. doi: 10.1371/journal.pone.0124227 (PMC4408046; doi:10.1371/journal.pone.0124227)
Supplement: S7 Table — (Reference Fig 9A). (PDF) [file pone.0124227.s007.pdf]

S7 Table: AST. (Reference Fig. 9A)

**AST (IU/L)dataset**						
Treatment (Tx)						
Pig#	BSL	6h	12h	24h	1wk	1mo
91079	48	228	502	612	58	33
91080	31	143	108	84	48	99
91081	36	249	541	529	96	27
91082	39	99	129	118	76	31
91084	26	34	50	78	27	18
91085	23	218	215	163	58	28
91086	22	113	114	92	24	21
90959	20	94	100	68	16	20
90962	45	67	51	50	35	47
Average Tx Group (n=9)	32.22222	138.3333	201.1111	199.3333	48.66667	36
Std Deviation Tx Group	10.31719	76.47222	188.1824	213.8931	26.20592	25.18432
Control (Ctrl)						
Pig#	BSL	6h	12h	24h	1W	1M
(Ctrl) 91083	38	104	124	102	43	20
(Ctrl) 90960	28	265	333	383	29	20
(Ctrl) 90961	60	152	377	389	36	36
(Ctrl) 90963	65	251	660	994	75	28
(Ctrl) 90964	41	118	139	129	30	19
Average Control Group (n=5)	46.4	178	326.6	399.4	42.6	24.6
Std Deviation Control Group	15.56599	75.24958	217.9319	358.9879	18.95521	7.334848
