# Supplementary material for: Safety of Intracoronary Infusion of 20 Million C-Kit Positive Human Cardiac Stem Cells in Pigs
Source: PLoS One. 2015 Apr 23;10(4):e0124227. doi: 10.1371/journal.pone.0124227 (PMC4408046; doi:10.1371/journal.pone.0124227)
Supplement: S8 Table — (Reference Fig 9B). (PDF) [file pone.0124227.s008.pdf]

S8 Table: Total CPK. (Reference Fig. 9B)

**Total CPK (IU/L) dataset**						
Treatment (Tx)						
Pig#	BSL	6h	12h	24h	1wk	1mo
91079	532	12487	36129	35886	641	652
91080	463	8658	6940	4412	739	1275
91081	609	15328	24006	17942	1271	430
91082	699	5160	7641	6967	1179	614
91084	498	1535	1767	1138	382	483
91085	531	20750	14789	12580	920	556
91086	515	9383	6055	5343	332	652
90959	454	13122	10738	6180	357	823
90962	825	5175	3952	2792	572	540
Average Tx Group (n=9)	569.5556	10177.56	12446.33	10360	710.3333	669.4444
Std Deviation Tx Group	122.0984	5917.585	11086.95	10873.39	349.7328	253.8898
Control (Ctrl)						
	BSL	6h	12h	24h	1W	1M
(Ctrl) 91083	1136	10606	9540	7282	774	1162
(Ctrl) 90960	1453	18828	32892	40997	854	670
(Ctrl) 90961	1309	13393	25135	21285	556	541
(Ctrl) 90963	1232	19205	48888	59850	1279	486
(Ctrl) 90964	969	6533	7082	7370	352	481
Average Control Group (n=5)	1219.8	13713	24707.4	27356.8	763	668
Std Deviation Control Group	181.9415	5422.95	17266.85	22810.08	348.722	286.4708
