# Supplementary material for: Safety of Intracoronary Infusion of 20 Million C-Kit Positive Human Cardiac Stem Cells in Pigs
Source: PLoS One. 2015 Apr 23;10(4):e0124227. doi: 10.1371/journal.pone.0124227 (PMC4408046; doi:10.1371/journal.pone.0124227)
Supplement: S9 Table — (Reference Fig 9C). (PDF) [file pone.0124227.s009.pdf]

S9 Table: Alk Phos. (Reference Fig. 9C)

**Alk Phos (IU/L) dataset**						
Treatment (Tx)						
Pig#	BSL	6h	12h	24h	1W	1M
91079	114	185	180	163	95	74
91080	120	158	152	136	105	89
91081	125	179	187	158	96	125
91082	185	239	218	164	119	160
91084	146	163	164	134	135	109
91085	160	210	223	180	113	152
91086	116	139	153	138	105	105
90959	139	176	205	189	150	162
90962	161	226	204	193	149	104
Average Tx Group (n=9)	140.6667	186.1111	187.3333	161.6667	118.5556	120
Std Deviation Tx Group	24.43358	32.89546	26.99074	22.55549	21.35481	31.80409
Control (Ctrl)						
	BSL	6h	12h	24h	1W	1M
(Ctrl) 91083	121	162	154	139	131	139
(Ctrl) 90960	118	180	156	144	124	122
(Ctrl) 90961	134	200	184	134	130	93
(Ctrl) 90963	114	172	197	172	111	97
(Ctrl) 90964	138	165	159	146	111	124
Average Control Group (n=5)	125	175.8	170	147	121.4	115
Std Deviation Control Group	10.44031	15.20526	19.352	14.73092	9.864076	19.45508
