# Supplementary material for: Safety of Intracoronary Infusion of 20 Million C-Kit Positive Human Cardiac Stem Cells in Pigs
Source: PLoS One. 2015 Apr 23;10(4):e0124227. doi: 10.1371/journal.pone.0124227 (PMC4408046; doi:10.1371/journal.pone.0124227)
Supplement: S10 Table — (Reference Fig 9D). (PDF) [file pone.0124227.s010.pdf]

S10 Table: ALT. (Reference Fig. 9D)

**ALT (IU/L) dataset**						
Treatment (Tx)						
Pig#	BSL	6h	12h	24h	1W	1M
91079	31	44	67	97	64	38
91080	34	41	46	50	35	45
91081	40	56	77	95	55	38
91082	50	53	53	53	43	33
91084	25	27	31	33	36	22
91085	36	55	69	81	53	32
91086	36	113	51	51	40	39
90959	25	33	44	38	24	24
90962	52	61	58	59	56	51
Average Tx Group (n=9)	36.55556	53.66667	55.11111	61.88889	45.11111	35.77778
Std Deviation Tx Group	9.593111	24.93492	14.27799	23.57671	12.73229	9.270623
Control (Ctrl)						
Pig#	BSL	6h	12h	24h	1W	1M
(Ctrl) 91083	40	49	57	61	47	58
(Ctrl) 90960	30	47	54	62	53	34
(Ctrl) 90961	38	38	54	68	47	32
(Ctrl) 90963	30	41	69	105	70	32
(Ctrl) 90964	30	33	40	42	34	30
Average Control Group (n=5)	33.6	41.6	54.8	67.6	50.2	37.2
Std Deviation Control Group	4.97996	6.542171	10.32957	23.07163	13.06522	11.71324
