# Supplementary material for: Safety of Intracoronary Infusion of 20 Million C-Kit Positive Human Cardiac Stem Cells in Pigs
Source: PLoS One. 2015 Apr 23;10(4):e0124227. doi: 10.1371/journal.pone.0124227 (PMC4408046; doi:10.1371/journal.pone.0124227)
Supplement: S1 Table — (Reference Fig 4). (PDF) [file pone.0124227.s001.pdf]

S1 Table: c-kit positivity and cell viability. (Reference Fig. 4)

Cell line	1	2	3	4	5	6	7	Mean	SD
% c-kit+	90.3	80.6	83.3	85.3	93.2	87.4	78.9	85.571	5.137
SEM								1.94	
Cell Line	1	2	3	4	5	6	7	Mean	SD
Viability %	98	99	97	98	98	96	99	97.857	1.069
SEM								0.9	
